# Retrospective Parameter Estimation and Forecast of Respiratory Syncytial Virus in the United States

**DOI:** 10.1371/journal.pcbi.1005133

**Published:** 2016-10-07

**Authors:** Julia Reis, Jeffrey Shaman

**Affiliations:** Department of Environmental Health Sciences, Mailman School of Public Health, Columbia University, New York, New York, United States of America; University of Texas at Austin, UNITED STATES

## Abstract

Recent studies have shown that systems combining mathematical modeling and Bayesian inference methods can be used to generate real-time forecasts of future infectious disease incidence. Here we develop such a system to study and forecast respiratory syncytial virus (RSV). RSV is the most common cause of acute lower respiratory infection and bronchiolitis. Advanced warning of the epidemic timing and volume of RSV patient surges has the potential to reduce well-documented delays of treatment in emergency departments. We use a susceptible-infectious-recovered (SIR) model in conjunction with an ensemble adjustment Kalman filter (EAKF) and ten years of regional U.S. specimen data provided by the Centers for Disease Control and Prevention. The data and EAKF are used to optimize the SIR model and i) estimate critical epidemiological parameters over the course of each outbreak and ii) generate retrospective forecasts. The basic reproductive number, *R*_*0*_, is estimated at 3.0 (standard deviation 0.6) across all seasons and locations. The peak magnitude of RSV outbreaks is forecast with nearly 70% accuracy (i.e. nearly 70% of forecasts within 25% of the actual peak), four weeks before the predicted peak. This work represents a first step in the development of a real-time RSV prediction system.

## Author Summary

Respiratory syncytial virus (RSV) is the most common cause of acute lower respiratory infection and bronchiolitis. Prompt treatment of RSV is necessary to prevent damage to lung tissue, complications from prolonged oxygen deprivation, and the potential development of reactive airway disorders. Another respiratory disease, influenza, has been simulated and forecast with high accuracy, using an epidemiological model and data assimilation methods. Here, we adapt such a model-filter system to simulate and forecast RSV epidemics in the United States. We find that the timing and volume of RSV epidemics can be forecast with high accuracy. Advance warning of the epidemic timing and volume of RSV patients has the potential to help medical centers prepare for a surge in infected patients and thus reduce delays to treatment in emergency departments.

## Introduction

Respiratory Syncytial Virus (RSV) infects nearly all children within the first two years of life, and is the most common cause of respiratory infection and bronchiolitis requiring hospitalization for infants under the age of one [1]. The rate of hospitalization for RSV infection, estimated at 1.3–3% for children under four [2,3], is greatest in infants younger than three months old [4], whose immune response can inundate and block smaller airways, leading to wheezing and difficulty breathing. Infants at risk for developing severe RSV infection are injected with the antibody prophylaxis palivizumab each month prior to anticipated RSV exposure. Palivizumab is highly rationed, primarily because of its high cost, so the scheduling of immunoprophylaxis is very important [5]; however, even though RSV activity cycles seasonally, the precise timing and magnitude of RSV incidence varies each year. This variability not only complicates effective infant dosing of immunoprophylaxis but also may contribute to well-documented delays in emergency departments (ED) [6]. RSV infections constitute 2% of hospital patients under age one on an annual basis [7] and often coincide with the influenza season.

The toll of RSV on the elderly is also high. RSV hospitalization rates in New York City are 1.5 times greater for individuals 75 and older than for 1-4-year-olds [7]. Older patients account for 25% of all ED visits [8], and as populations in the developed world age, the proportion of elderly in the ED, some infected with RSV, is likely to grow. Indeed, ED crowding has been linked to delays in treatment and reduced efficacy of care for asthma and respiratory distress [9]. Prompt administration of oxygen is key to the treatment of bronchiolitis, although the minimum level of oxygen saturation (SO_2_) recommended for children varies from 90%–95% [10–13]. Children with prolonged oxygen deprivation in the range 90%–94% SO_2_, due to a variety of causes, were found to be at risk for long-lasting cognitive damage and associated behavioral issues [14–16]. The immune response to bronchiolitis can in some cases damage lung tissue [17,18]. Accurate forecasts of RSV incidence could thus be used by health care management to develop data-driven strategies that are more effective and better timed for patient demand [19,20]. Here we present the development and validation of a model-inference system for forecasting the timing and magnitude of RSV outbreaks.

The core component of our forecasting system is a dynamic model describing the propagation of RSV through a simulated population. Many of the dynamic models previously developed for simulation of RSV are compartmental constructs that divide a population into the different states associated with the chain of infection: susceptible, infected, and recovered (SIR), as well as constructs additionally accounting for partial immunity (SIRS) and exposure (SEIRS) [21–23]. Epidemiological parameters within these model structures govern the rates of transition between compartments. Typically, such parameters are either designated *a priori* based on clinical and laboratory estimates, or estimated from historical incidence, using a compartmental model structure and transmission dynamics. RSV parameter estimates derived using these different methods or data, however, can vary widely. For example, the parameter that represents the duration of infection, *D*, has been identified variously through antigen detection as 6.7 days [24], using RT-PCR assay as 11.2 days [25] and as nearly three months in immunocompromised individuals [26]. Similarly, for the basic reproductive number, *R*_*0*_, one study in Florida found mean *R*_*0*_ values of 1.7 and 7.4 using an SIRS model and modified SEIRS model, respectively [21], while others in the same state found a mean *R*_*0*_ above 9 using a SIRS that accounted for repeated infections, waning immunity, and/or age structure [22,23]. Much of this variation reflects the use of different model structures, and in part it may stem from the quality, abundance, and spatial and temporal resolution of the RSV data used to make these inferences.

Here, the same model-inference system developed for the generation of RSV forecasts is also used to infer critical RSV epidemiological parameters. We apply this system at a broad scale using regional RSV data from the U.S. and in so doing attempt to further resolve RSV parameter estimates. To perform this work, and as has been performed for other infectious disease systems [27–29], we combine a simple dynamical disease transmission model with a data assimilation filter that updates the model state variables and parameters using time series observations. Here, we present this RSV model-filter system, the resulting parameter estimates, and retrospective forecasts generated for the United States. We have built this predictive system for a variety of RSV data streams. It can be deployed at the beginning of the annual RSV wintertime season and used to help reduce delays in health care and enable broader access to preventative medication.

## Materials and Methods

### Data

RSV specimen data were provided by the Centers for Disease Control and Prevention (CDC). These data, sampled via antigen detection, viral isolation, and polymerase chain reaction, are collected and administered by laboratories and other medical facilities that participate in the National Respiratory and Enteric Virus Surveillance System (NREVSS). The number of samples tested for RSV each week and the number of positive results were recorded, since July 2004 at the census division and Health and Human Services (HHS) region geographic scales (the states in each regional classification system are listed in S1 and S2 Tables). The RSV season runs from week 27 (around July 10^th^) through week 26 of the following year, with at least 20 tests per week during this season. Over the first ten weeks of the RSV season, incidence is low and testing is often limited; consequently, we omitted these first ten weeks and began all simulations on week 37, in mid-September, when the number of RSV tests administered typically begins to rise. To scale the number of positive tests to regions with very different reporting magnitudes, we divide by the number of laboratories reporting to each region each season. With these data, we estimated RSV incidence over nine census divisions, ten HHS regions, and ten RSV seasons. Census division RSV is presented in S1 Fig, along with a histogram of actual peak timing (S2 Fig).

### Dynamical Model, Filter, and Forecasting System

#### Model

The RSV data used in this study are not resolved by age. As a consequence, we use a simpler, perfectly-mixed compartmental model to describe RSV transmission dynamics. Furthermore, each RSV location and season is simulated and forecast independently. While reinfection with RSV is common, RSV antibodies only decline by 25–30% per year [30], indicating that waning immunity and consequent reinfection within the time frame of a single season is not likely to be significant [3,31]. Consequently, we chose to work with an SIR rather than an SIRS model. We used the following model form:

$$\frac{dS}{dt}=-\frac{R_{0}\;IS}{DN}$$ (1)

$$\frac{dI}{dt}=\frac{R_{0}\;IS}{DN}-\frac{I}{D}$$ (2)

where *S* is the susceptible population, *I* is the number of infected, *R*_0_ is the basic reproductive number, *D* is the mean infection period, and *N* is the population, which is held constant at an arbitrary size of 500,000 people. For all simulations, a 300-member ensemble was integrated in conjunction with data assimilation methods (see description below). For each ensemble member, the initial combination of model state variables and parameters was randomly selected from prescribed ranges using Latin hypercube-generated uniform distribution sampling (S3 Table).

#### Mapping data to number infected

To estimate RSV in the population model, we mapped the positive specimen data divided by the number of contributing laboratories, which is a passive sample of positive RSV tests among persons seeking medical care for RSV, to the incidence of RSV infections in the total model population. Specifically, we used a scaling factor, *γ*, to map the RSV data to incidence data in the population, *N*, i.e.:

$$p(RSV)=N\frac{p(g)}{p(g|RSV)}p(RSV|g)≅\gamma \;RSV$$ (3)

Here, *p(RSV)* is the probability of an RSV infection (RSV incidence in the SIR model), *p(g)* is the probability of access to RSV testing among the entire population, *p(RSV|g)* is the probability that someone with access to RSV testing is infected with RSV, and *p(g|RSV)* is the probability of access to a RSV testing facility if a person is infected with RSV. As shown in Eq 3, *γ* is defined as *p(g)/ p(g|RSV)*. We experimented with a range of possible values and selected a single value for simplicity, *γ* = 0.001, that produces RSV forecasts with the lowest RMSE errors.

#### Filter

We used the ensemble adjustment Kalman filter (EAKF) [32–34] to assimilate RSV data into the SIR model [35]. Starting from a 300-member ensemble of randomly initialized parameter values (S3 Table), we integrated this ensemble of SIR simulations forward in time to the first observation of the season. This model-generated estimate, of both the observed infections and the unobserved state space variables and parameters, is the Bayesian prior. The EAKF is then used to update these state variable and parameter estimates for all ensemble members, thereby generating a posterior. The ensemble is then integrated forward again to the next observation and the update process is repeated. Through this iterative process of integration and update, the ensemble of simulations is optimized by the EAKF [32].

### Calibration and Simulation

The random initialization of state variables and parameters for each 300-member ensemble simulation adds an element of stochastic variation to the otherwise deterministic EAKF. To account for any effects of initialization, each 300-member ensemble simulation was repeated ten times, in each instance with a new random selection of initial state variables and parameters. We explored the sensitivity of the model-inference system to other components of the system, including the scaling factor γ (as described above), observational error variance (OEV), the use of inflation, and range of initial state variables.

The EAKF uses OEV to describe uncertainty in the observed data, and weighs it against the variance of the SIR model simulations, which is estimated directly from the spread of the 300 ensemble simulations. More specifically, OEV is used by the EAKF to weigh the observations (RSV) and model prior estimation of RSV incidence, and to produce the model posterior estimation of both the state variables and parameters. As for influenza, the OEV for RSV was assumed to be small when observations over the prior 3 weeks ($\bar{OBS}$) had been small and to increase as observed RSV increased. Specifically,

$$OEV={OEV}_{0}+\frac{{\bar{OBS}}^{2}}{a}$$ (4)

where *OEV*_*0*_ is a minimum OEV and *a* is a constant. This form represents the increasing uncertainty of clinical data as epidemic infectiousness increases [32]. In vetting the RSV model-inference system we tested a range of values for *OEV*, and based on lowest RMSE, selected *OEV*_*0*_ and *a*, respectively, as 10^4^ and 50.

The EAKF successively adjusts the ensemble of simulations and in so doing iteratively reduces the variance of the ensemble. Should this variance become too small, the EAKF algorithm puts too much weight on the simulations and ignores the observations. As a consequence, the simulations begin to ‘diverge’ from the truth. To counteract this divergence, an inflation algorithm can be applied that increases the ensemble variance a small amount prior to each filter update. We applied a type of inflation called multiplicative inflation [34,36] and tested whether it improved forecast accuracy; however, we found that divergence was not an issue for our RSV simulations and that forecast accuracy was highest without inflation. We therefore present results for simulations without inflation.

In all we tested over 600 unique combinations of the scaling factor, inflation, OEV, and the initial state variable and parameter ranges for each location and year. S3 Fig presents the RMSE between observations and forecasts from four of these combinations. With the selected combination, we ran simulations for the nineteen (overlapping) locations and ten years, with ten repetitions of each unique combination to account for stochastic effects. Combined results from using both CD and HHS regions are presented unless otherwise noted.

#### Parameter estimation and forecast

We present *D* and *R*_*0*_ estimates from three time points during each outbreak: the epidemic peak estimated by the model-filter posterior, two weeks after the peak, and on the last week of the simulation time period. Retrospective forecasts were generated for 20 weeks (week 43 in late October to week 9 in early March) for each location and year by integrating the latest posterior of updated variables and parameters through to the end of the season. Forecasts were evaluated relative to lead week, defined here as the week of forecast minus the predicted peak week of the ensemble mean trajectory (i.e. negative lead weeks indicate that incidence will peak in the future).

We quantified forecast accuracy using four metrics: i) prediction of RSV outbreak peak timing, i.e. whether the ensemble mean trajectory prediction of peak timing is within ±1 week of the actual peak, ii) prediction of RSV outbreak peak intensity, i.e. whether the ensemble mean trajectory predicted peak outbreak incidence is within ±15% of observed peak incidence (with the observed as the denominator), iii) prediction of total RSV attack rate, i.e. whether the ensemble mean trajectory prediction of the seasonal attack rate, defined as the total number of cases per year, is within ±15% of observation, and iv) prediction of the onset of the RSV epidemic. We define the RSV onset at a threshold of γΝ for two consecutive weeks, which roughly corresponds to the CDC defined onset of 10% percent positive RSV cases for two consecutive weeks. For sensitivity, timing and onset each within ±2 weeks and magnitude and attack rate each within ±25% are also presented.

These prediction metrics were also evaluated to determine how forecast accuracy varied as a function of the ensemble variance [32,35]. Here, we explored whether forecast accuracy improved as the variance among the 300 ensemble members decreased. Such relationships can be used to ascribe certainty to a given forecast as it is produced in real time.

## Results

### Simulation of the RSV

**Fig 1:**
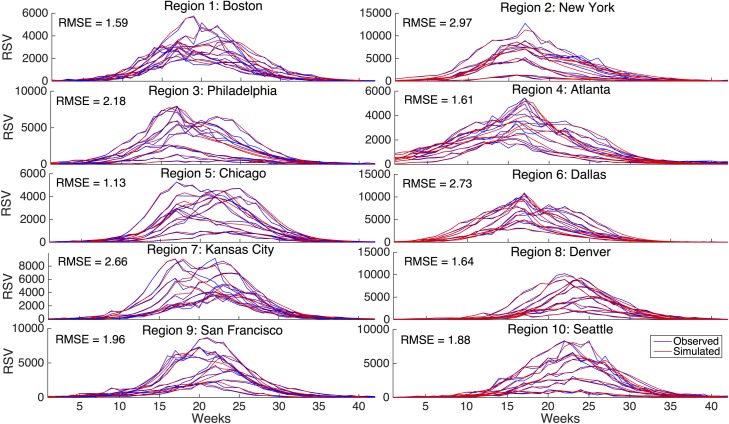
The historical time series and model-filter simulations of RSV, for each HHS region in the United States over ten RSV seasons (off-season weeks were not modeled). The mean RMSE between the observed and simulated scaled data per region over all ten seasons, divided by γN, is shown in each subplot.

Our simulations of RSV time series using the model-filter system replicated the historical data well. Fig 1 presents the historical time series and model-filter simulations for RSV at the HHS regional classification system over ten seasons (2004–2005 through 2013–2014). The mean RMSE between simulated and observed scaled data over all ten seasons, in both geographical groupings, ranged from 1.08 to 2.97 (Fig 1 and S4 Fig).

### Estimation of Epidemiological Parameters

**Fig 2:**
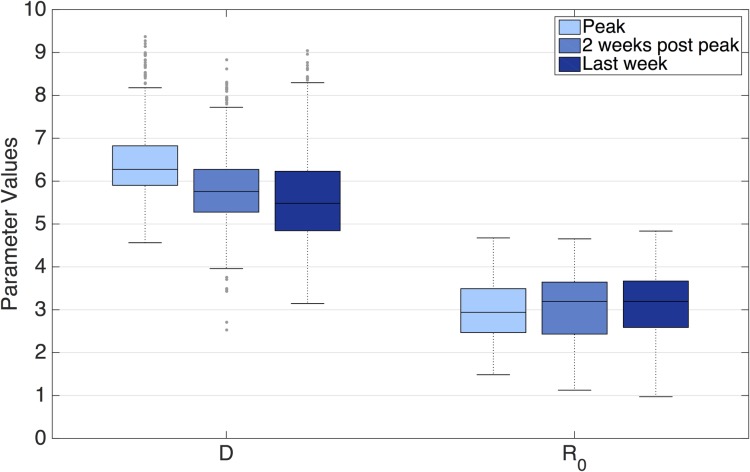
A boxplot of ensemble mean estimates for epidemiological parameters *D* (days) and *R*_*0*_ at the observed peak, 2 weeks after the peak, and on the last week of simulation for all seasons and regions. The central line indicates the median, the box provides the interquartile range, and the whiskers give the extrema (outliers are shown, defined as 1.5 times greater the interquartile range).

Ensemble mean estimates of the epidemiological parameters *D* and *R*_*0*_ for RSV at outbreak peak are shown in Fig 2. At the simulated epidemic peak, the mean values of *D* and *R*_*0*_ were 6.4 days and 3.0, respectively. During the period of forecast, there is little variation of these estimates, though the minimum, 5^th^ percentile, 95^th^ percentile, and maximum parameter values vary more (S5 Fig). *R*_*0*_ shows some geographic variability and is consistently lower in Census Division 5 (mean *R*_*0*_ = 2.5), which stretches from Florida to Maryland (S6 and S7 Figs). Depending on season and location, *D* and *R*_*0*_ vary between about 5–9 and 1.5–4, respectively (S7 Fig). Estimates of the parameter *D*, but not *R*_*0*_, were found to be sensitive to γ (S8 Fig).

### Retrospective Forecasts

**Fig 3:**
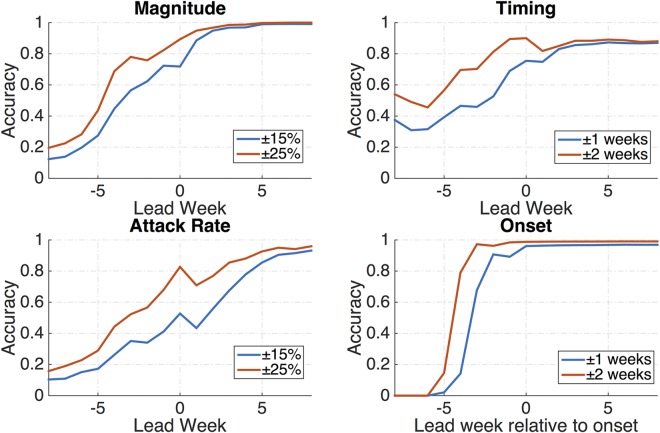
The fraction of RSV forecasts accurate for prediction of peak magnitude, peak timing, attack rate, and onset. Peak magnitude, peak timing, and attack rate are shown as a function of the predicted peak timing lead (current week minus predicted peak); onset is shown relative to predicted onset lead.

Fig 3 presents overall forecast accuracy as a function of lead week for RSV. Lead week is defined as the current week minus the predicted peak week, and onset accuracy is presented as the current week minus the onset week. For both regional groupings, the ensemble forecasts of peak magnitude have nearly 70% accuracy (within ±25%) with a four-week lead-time, and nearly 70% accuracy (±1 week) in forecasting the onset week with a three-week lead (Fig 3). Forecasts of epidemic peak timing (±1 week) are correct with over 68% accuracy with a one-week lead-time. Forecasts of epidemic peak timing are within ±2 weeks of the observed peak 81% of the time with a two-week lead-time. Forecasts of the seasonal attack rate are accurate 89% of the time within ±25% of the observed, with a one-week lead-time. Sample forecasts are shown in S9 and S10 Figs, and a boxplot presents peak timing accuracy over all ten years for both regions (S11 Fig).

### Forecast Calibration

**Fig 4:**
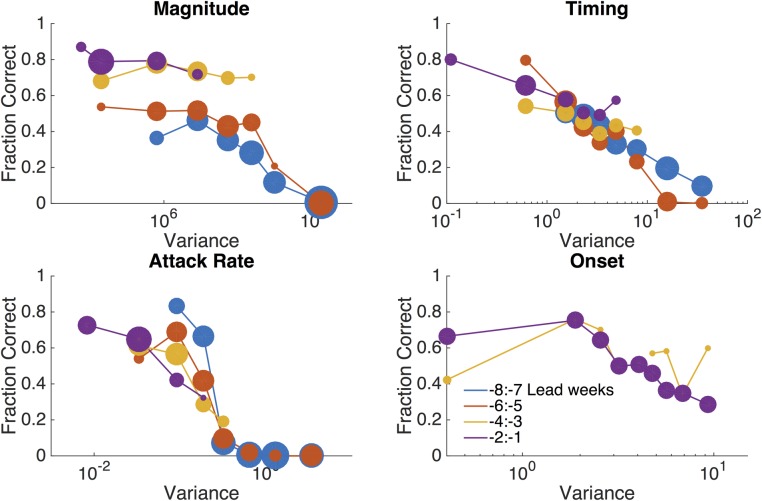
The fraction of RSV forecasts accurately predicting peak timing (±1 week), magnitude (±25%), attack rate (±25%), and onset (±1 week), shown as a function of ensemble variance. Forecasts are stratified by lead week, defined as current week minus predicted peak. The marker size is scaled to the number of runs in each variance bin.

To generate calibrated forecasts, we used ensemble variance to infer the expected accuracy of predicted outcomes [35]. Forecast accuracy for each metric is plotted versus ensemble variance, grouped by lead week (Fig 4). Variance is binned by percentile intervals of 10% (making ten bins), and only bins containing at least 70 runs are shown. For the onset criterion, variance is computed only from forecasts that begin before the onset. These plots show that forecast accuracy for all four metrics generally increases as ensemble variance decreases. That is, as within-ensemble agreement rises, the forecasts are more accurate. These relationships can be used to infer the expected accuracy of a real-time forecast. Further evaluation of forecast accuracy as a function of week of forecast and lead week is also presented (S12 Fig).

**Fig 5:**
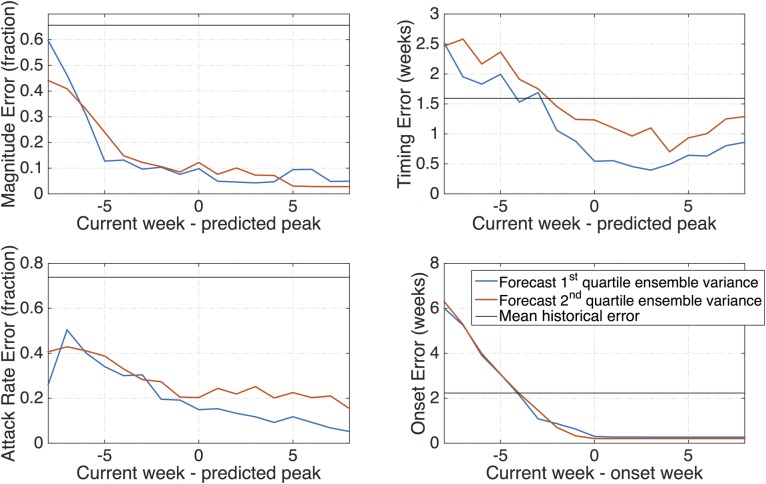
Mean absolute error for each criterion as a function of lead week. The model-filter error is shown for the 1^st^ and 2^nd^ lowest quartiles of ensemble variance, and the historical mean forecast error is shown for comparison.

Fig 5 presents the relative absolute error of magnitude and attack rate, and the absolute error for timing and onset. Error for the lowest two quartiles of ensemble variance is plotted. Forecast accuracy exceeds the mean historical error for magnitude and attack rate at least eight weeks before the predicted peak. The model-filter forecasts exceed the historical mean peak timing prediction more than two weeks before the predicted peak for the lowest two quartiles, and the onset prediction exceeds the historical mean at 4 weeks lead for the lowest quartiles (Fig 5). For onset, this 50^th^ percentile corresponds to a mean ensemble variance of 3.8, meaning that model-filter forecasts with this variance or below should be trusted over the historical mean when predicting onset four weeks in the future. A scatter plot shows the model-filter forecast error subtracted from the historical mean forecast plotted as a function of observed regional RSV standard deviation for peak magnitude, peak timing, attack rate, and onset (S13 Fig). This figure, which includes only forecasts falling in the lowest 50^th^ ensemble variance, shows that in regions with greater year-to-year RSV variability, the model-filter forecasts increasingly outperform historical expectance. Linear regressions for each criterion and lead week grouping are also plotted on S13 Fig. S4 Table provides these regression slopes and intercepts and their statistical significance. S4 Table could be used to decide between the historical mean and model-filter forecasts, based on the region’s standard deviation.

## Discussion

Many of the state-of-the-art forecast systems developed for respiratory pathogens such as influenza use ensemble predictions generated with a mathematical model of disease transmission, such as an SIR model, that has been optimized using Bayesian inference methods [27,33,35,37–39]. These inference methods enable estimation of model state variables (e.g. number of susceptible and infected) and critical epidemiological parameters (e.g. *R*_*0*_). Here we paired an SIR model with the EAKF to predict RSV incidence. We have shown that the model-filter forecasts are generally more accurate than historical expectance (Fig 5), and that forecast accuracy can be distinguished using ensemble variance (Fig 4). This latter finding enables distinction of forecast reliability in real time.

The improvement over historical expectance is particularly important. The scaled RSV positive data used in this study varies in magnitude substantially from year-to-year; however, the observed mean error for peak timing is only about 1.5 weeks and thus fairly regular, allowing reasonable forecast with the historical mean. Still the model-filter forecasts exceed the accuracy of the historical mean more than two weeks prior to the predicted peak (Fig 5). In addition, the improvement of the model-filter forecasts over the historical mean predictions, as measured by the difference in error, is correlated with observed standard deviation within a region (S13 Fig); for example, model-filter forecasts of epidemic peak magnitude provide a greater reduction of error in regions with greater standard deviation of peak magnitude. Thus, regions with greater observed variability see a greater improvement of forecast accuracy from the model-filter system over historical expectance.

RSV patients in medical care are overwhelmingly the very young and the elderly, because of the small size of their lungs or less-effective immune response [40,41]. Yet despite this bimodal age distribution of apparent RSV infection, asymptomatic healthy adults can transmit RSV [25,42]. As the NREVSS data do not include age-stratified infection, we were unable to simulate these differences and instead used a single age class model. Should age-stratified infection data become available, future work should explore forecast and parameter estimation with an age-stratified model. Prior work with models that account for age structure, tend to produce higher estimates of *R*_*0*_ than simpler SIR or SIRS models [21–23]. Our parameter estimates reflect our use of a simpler model structure and produce estimates similar to those described by single-season SIR [43] and SIRS [21] modeling efforts.

Our data regions include up to eight states with distinct climates. Assignment of states to regions differs between the census division and HHS designations (S1 and S2 Tables), though there exists considerable overlap. Forecast accuracy did not differ appreciably between census divisions and HHS regions. By simulating both regional grouping systems, we further demonstrate the reliability of our results. State actors could consider forecasts from both regional classification systems, particularly for regions including Florida, a populous state in which typically RSV peaks earlier than other states.

For our results, *R*_*0*_ is estimated with a mean of 3.0, which is within the range of 1.2 to over 9 identified using other dynamical models [21–23,43]. Our estimate no doubt reflects the quality and geographic scale of the RSV data, as well as the model structure. The mean value of *D*, the duration of infection, just over 6 days, is also within the range modeled variously as 5–11 days. In our SIR model, the duration of infection functions as the duration of infectiousness, which in our model-filter may vary by time or by region depending on norms such as the time to treatment or isolation of symptomatic individuals. We found that the duration of infection, *D*, is somewhat sensitive to the choice of γ (S8 Fig). Clinical and experimental data have yet to identify the viral load needed for significant RSV transmission. These information are needed to better constrain the mean duration of infectivity. Once this is more definitively established, *D* could be fixed while other parameters are estimated by the EAKF.

More doctors and researchers are simultaneously testing for multiple viruses including RSV, using PCR-based panel assays; yet, as July of 2014, the American Academy of Pediatrics no longer recommends testing bronchiolitis patients for RSV, because treatment is the same regardless of etiology. Without widespread RSV testing, the efficacy of an RSV vaccine, several of which are currently under development [44], may be more difficult to determine. Further, testing would support both study and forecast of RSV. For example, RSV data derived from testing of bronchiolitis patients and active sampling, the latter used to estimate RSV incidence across all age groups, would support development of more complicated model structures, which could be used to generate age-stratified forecasts. Ideally, these forecasts would influence individual behavior and improve health care preparedness, and thus would serve as a bulwark against bronchiolitis.

In summary, advanced warning of RSV infections can reduce delays in the care of RSV patients, who are predominantly infants and the elderly, and hence vulnerable to lingering damage to lung tissue and other negative outcomes from RSV. Our model-filter system replicates the seasonal dynamics of RSV throughout the United States, and our forecasts predict multiple outcomes, including epidemic peak magnitude with more than 60% accuracy five weeks prior to the predicted peak. Importantly, the high accuracy of forecasts predicting epidemic onset one month in advance could allow doctors to better ration the delivery of palivizumab prophylaxis. Further, the near-linear increase in accuracy with decreasing ensemble variance provides a metric of certainty for each forecast. Our results provide evidence that levels of RSV incidence can be anticipated in time to inform the distribution of prophylactics and deployment of other protective measures against RSV infection. However, real-time predictions will require that laboratory testing for RSV be resumed.

## Supporting Information

S1 Fig Scaled RSV for the nine U.S. Census Divisions and ten RSV seasons. (TIF)

S2 Fig A histogram of actual peak timing for each census division and year. (TIF)

S3 Fig The overall RMSE between the observations and forecasts shown for two γ and OEV combinations. The variable OEV was computed with Eq 4, with *OEV*_*0*_ and *a* equal to 10^4^ and 50, respectively.
(TIF)

S4 Fig Scatterplot of simulated RSV infections versus observed infections for the 9 census divisions and all 10 seasons, multiplied by γN as described in the text. The red line is 1:1.
(TIF)

S5 Fig Estimates of the parameters *R*_*0*_ and *D* shown as a time series during the 20 weeks of forecast. All regions and seasons are shown.
(TIF)

S6 Fig Boxplots of weekly *R*_*0*_ estimates for each census division across all 10 seasons. (PDF)

S7 Fig The mean values of *D* and *R*_*0*_ for RSV for each year and census division. These estimates remain similar from year to year and location. Overall, the smallest mean *R*_*0*_ is 2.5, in Census Division 5, which contains Florida, and the largest mean *R*_*0*_ is 3.4 in Census Division 4.
(TIF)

S8 Fig Boxplots of ensemble mean estimates for the parameters *D* and *R*_*0*_ at the epidemic peak using RSV and γ = 0.00075 and 0.0001. (TIF)

S9 Fig RSV observed (O) and forecasts for 2007–2008, for each census division. Each forecast shown is the mean of 300 ensemble members. The thick black line is observed RSV. The thin colored lines represent the ensemble mean trajectories of successive weekly forecasts, beginning Week 43 through Week 10.
(TIF)

S10 Fig RSV observed (Obs) and forecasts for 2011–2012, for each census division. Each forecast shown is the mean of 300 ensemble members. The thick black line is observed RSV. The thin colored lines represent the ensemble mean trajectories of successive weekly forecasts, beginning Week 43 through Week 10.
(TIF)

S11 Fig Peak timing forecast accuracy (for both census division and HHS regions) over the ten seasons of RSV epidemics. All forecasts, regardless of ensemble variance, are shown.
(TIF)

S12 Fig The error of RSV forecast for magnitude, timing error, attack rate, onset for each week of forecast and lead week. Blanks indicate week and lead week combinations without forecasts. Error decreases as both week of forecast and lead week increase. Including week of forecast along with ensemble variance and lead week could refine prediction calibration.
(TIF)

S13 Fig Historical mean prediction error minus model-filter forecasts error, plotted by region as a function of regional RSV standard deviation, and grouped by lead week. Scatter points above the zero line represent forecasts that outperform the historical mean. Both census division (CD) and HHS regions are plotted. Only forecasts with ensemble variance in the bottom 50^th^ percentile, taken over the entire forecast period for each region, are shown; during some lead weeks, there are no forecasts in the bottom 50^th^ percentile, hence some regions do not have all four lead week groupings (e.g. 7–8 weeks before the predicted onset). Positive significant linear correlations (alpha = 0.05) between difference in error and observed standard deviation are found for each forecast criterion at least four weeks in advance of the predicted peak or onset. S4 Table lists the regression line slope estimates for each regression and statistical significance.
(TIF)

S1 Table US geographic partitions comprising each of the 10 US Department of Health and Human Services regions. (DOCX)

S2 Table US states comprising each of the 9 US Census Divisions. (DOCX)

S3 Table State variables and parameters estimated using the SIRS-EAKF system and the range of values employed for random initialization. (DOCX)

S4 Table Statistics for the difference between the historical regional mean and model-filter forecasts error regressed upon the observed standard deviation of each criterion (plotted in S12 Fig). Only forecasts with ensemble variance in the lowest 50th percentile were included.
(DOCX)

## References

[1] Hall CB, Weinberg GA, Iwane MK, Blumkin AK, Edwards KM, Staat MA, et al The Burden of Respiratory Syncytial Virus Infection in Young Children. N Engl J Med. 2009;360: 588–598. 10.1056/NEJMoa0804877 19196675 PMC4829966

[2] Belshe RB, Van Voris LP, Mufson MA. Impact of Viral Respiratory Diseases on Infants and Young Children in a Rural and Urban Area of Southern West Virginia. Am J Epidemiol. 1983;117: 467–474. 6404161 10.1093/oxfordjournals.aje.a113564

[3] Glezen WP. Risk of Primary Infection and Reinfection With Respiratory Syncytial Virus. Arch Pediatr Adolesc Med. American Medical Association; 1986;140: 543 10.1001/archpedi.1986.02140200053026 3706232

[4] Stockman LJ, Curns AT, Anderson LJ, Fischer-Langley G. Respiratory Syncytial Virus-associated Hospitalizations Among Infants and Young Children in the United States, 1997–2006. Pediatr Infect Dis J. 2012;31: 5–9. 10.1097/INF.0b013e31822e68e6 21817948

[5] Panozzo CA, Stockman LJ, Curns AT, Anderson LJ. Use of Respiratory Syncytial Virus Surveillance Data to Optimize the Timing of Immunoprophylaxis. Pediatrics. 2010;126: e116–e123. 10.1542/peds.2009-3221 20547651

[6] Magid DJ, Asplin BR, Wears RL. The quality gap: Searching for the consequences of emergency department crowding. Ann Emerg Med. 2004;44: 586–588. 10.1016/j.annemergmed.2004.07.449 15573033

[7] Goldstein E, Greene SK, Olson DR, Hanage WP, Lipsitch M. Estimating the hospitalization burden associated with influenza and respiratory syncytial virus in New York City, 2003–2011. Influenza Other Respi Viruses. 2015;9: 225–233. 10.1111/irv.12325 25980600 PMC4548992

[8] Samaras N, Chevalley T, Samaras D, Gold G. Older Patients in the Emergency Department: A Review. Ann Emerg Med. 2010;56: 261–269. 10.1016/j.annemergmed.2010.04.015 20619500

[9] Sills MR, Fairclough D, Ranade D, Kahn MG. Emergency Department Crowding Is Associated With Decreased Quality of Care for Children With Acute Asthma. Ann Emerg Med. 2011;57: 191–200.e7. 10.1016/j.annemergmed.2010.08.027 21035903

[10] Poets CF. When do infants need additional inspired oxygen? A review of the current literature. Pediatr Pulmonol. 1998;26: 424–8. 10.1002/(SICI)1099-0496(199812)26:6<424::AID-PPUL7>3.0.CO;2-G 9888217

[11] Ralston SL, Lieberthal AS, Meissner HC, Alverson BK, Baley JE, Gadomski AM, et al Clinical practice guideline: the diagnosis, management, and prevention of bronchiolitis. Pediatrics. American Academy of Pediatrics; 2014;134: e1474–502. 10.1542/peds.2014-2742 25349312

[12] Walsh P, Rothenberg SJ. American Academy of Pediatrics 2014 Bronchiolitis Guidelines: Bonfire of the Evidence. West J Emerg Med. California Chapter of the American Academy of Emergency Medicine (Cal/AAEM); 2015;16: 85–8. 10.5811/westjem.2015.1.24930 25671015 PMC4307733

[13] Bronchiolitis in children: diagnosis and management: NICE guidelines [NG9]. NICE; 2015.

[14] Bass JL, Corwin M, Gozal D, Moore C, Nishida H, Parker S, et al The effect of chronic or intermittent hypoxia on cognition in childhood: a review of the evidence. Pediatrics. 2004;114: 805–816. 10.1542/peds.2004-0227 15342857

[15] Bass JL, Gozal D. Oxygen therapy for bronchiolitis. Pediatrics. 2007;119: 611 10.1542/peds.2006-3002 17332214

[16] Bass JL, Gozal D. Oxygen Therapy for Bronchiolitis: In Reply. Pediatrics. 2007;120 10.1542/peds.2007-1754 17332214

[17] Bem RA, Bos AP, Wösten-van Asperen RM, Bruijn M, Lutter R, Sprick MR, et al Potential Role of Soluble TRAIL in Epithelial Injury in Children with Severe RSV Infection. Am J Respir Cell Mol Biol. 2010;42: 697–705. 10.1165/rcmb.2009-0100OC 19635930

[18] Erickson S, Schibler A, Numa A, Nuthall G, Yung M, Pascoe E, et al Acute lung injury in pediatric intensive care in Australia and New Zealand–A prospective, multicenter, observational study. Pediatr Crit Care Med. 2007;8: 317–323. 10.1097/01.PCC.0000269408.64179.FF 17545931

[19] Welch SJ, Allen TL. Data-driven quality improvement in the Emergency Department at a level one trauma and tertiary care hospital. J Emerg Med. 2006;30: 269–276. 10.1016/j.jemermed.2005.07.007 16677976

[20] Stone-Griffith S, Englebright J, Cheung D, Korwek K, Perlin J. Data-driven process and operational improvement in the emergency department: the ED Dashboard and Reporting Application. J Healthc Manag. 2012;57: 167 22724375

[21] Weber A, Weber M, Milligan P. Modeling epidemics caused by respiratory syncytial virus (RSV). Math Biosci. 2001;172: 95–113. 10.1016/s0025-5564(01)00066-9 11520501

[22] Pitzer VE, Viboud C, Alonso WJ, Wilcox T, Metcalf CJ, Steiner C a., et al Environmental Drivers of the Spatiotemporal Dynamics of Respiratory Syncytial Virus in the United States. PLoS Pathog. 2015;11: e1004591 10.1371/journal.ppat.1004591 25569275 PMC4287610

[23] White LJ, Mandl JN, Gomes MGM, Bodley-Tickell AT, Cane PA, Perez-Brena P, et al Understanding the transmission dynamics of respiratory syncytial virus using multiple time series and nested models. Math Biosci. 2007;209: 222–39. 10.1016/j.mbs.2006.08.018 17335858 PMC3724053

[24] Hall CB, Douglas RG, Geiman JM. Respiratory syncytial virus infections in infants: Quantitation and duration of shedding. J Pediatr. 1976;89: 11–15. 10.1016/S0022-3476(76)80918-3 180274

[25] Munywoki PK, Koech DC, Agoti CN, Kibirige N, Kipkoech J, Cane PA, et al Influence of age, severity of infection, and co-infection on the duration of respiratory syncytial virus (RSV) shedding. Epidemiol Infect. 2015;143: 804–812. 10.1017/S0950268814001393 24901443 PMC4411640

[26] Schwarze J, O’Donnell DR, Rohwedder A, Openshaw PJM. Latency and persistence of respiratory syncytial virus despite T cell immunity. Am J Respir Crit Care Med. American Thoracic Society; 2004;169: 801–5. 10.1164/rccm.200308-1203OC 14742302

[27] Yang W, Cowling BJ, Lau EHY, Shaman J. Forecasting Influenza Epidemics in Hong Kong. Koelle K, editor. PLOS Comput Biol. 2015;11: e1004383 10.1371/journal.pcbi.1004383 26226185 PMC4520691

[28] Yang W, Karspeck A, Shaman J. Comparison of filtering methods for the modeling and retrospective forecasting of influenza epidemics. PLoS Comput Biol. Public Library of Science; 2014;10: e1003583 10.1371/journal.pcbi.1003583 24762780 PMC3998879

[29] Shaman J, Yang W, Kandula S. Inference and forecast of the current west african ebola outbreak in Guinea, sierra leone and liberia. PLoS Curr. 2014;6 10.1371/currents.outbreaks.3408774290b1a0f2dd7cae877c8b8ff6 25642378 PMC4234409

[30] Kutsaya A, Teros-Jaakkola T, Kakkola L, Toivonen L, Peltola V, Waris M, et al Prospective clinical and serological follow-up in early childhood reveals a high rate of subclinical RSV infection and a relatively high reinfection rate within the first 3 years of life. Epidemiol Infect. Cambridge University Press; 2016; 1–12. 10.1017/S0950268815003143 PMC9150639 26732801

[31] Belshe RB, Van Voris LP, Mufson MA. Impact of viral respiratory diseases on infants and young children in a rural and urban area of southern West Virginia. Am J Epidemiol. 1983;117: 467–74. 6404161 10.1093/oxfordjournals.aje.a113564

[32] Shaman J, Karspeck A, Yang W, Tamerius J, Lipsitch M. Real-time influenza forecasts during the 2012–2013 season. Nat Commun. Nature Publishing Group; 2013;4: 2837 10.1038/ncomms3837 24302074 PMC3873365

[33] Karspeck AR, Anderson JL. Experimental implementation of an ensemble adjustment filter for an intermediate ENSO model. J Clim. 2007;20: 4638–4658. 10.1175/JCLI4245.1

[34] Anderson JL. An Ensemble Adjustment Kalman Filter for Data Assimilation. Mon Weather Rev. 2001;129: 2884–2903. 10.1175/1520-0493(2001)129<2884:AEAKFF>2.0.CO;2

[35] Shaman J, Karspeck A. Forecasting seasonal outbreaks of influenza. Proc Natl Acad Sci. 2012;109: 20425–20430. 10.1073/pnas.1208772109 23184969 PMC3528592

[36] Whitaker JS, Hamill TM. Ensemble Data Assimilation without Perturbed Observations. Mon Weather Rev. 2002;130: 1913–1924. 10.1175/1520-0493(2002)130<1913:EDAWPO>2.0.CO;2

[37] Brooks LC, Farrow DC, Hyun S, Tibshirani RJ, Rosenfeld R. Flexible Modeling of Epidemics with an Empirical Bayes Framework. Tanaka MM, editor. PLOS Comput Biol. Public Library of Science; 2015;11: e1004382 10.1371/journal.pcbi.1004382 26317693 PMC4552841

[38] Ong JBS, Chen MI-C, Cook AR, Lee HC, Lee VJ, Lin RTP, et al Real-time epidemic monitoring and forecasting of H1N1-2009 using influenza-like illness from general practice and family doctor clinics in Singapore. PLoS One. Public Library of Science; 2010;5: e10036 10.1371/journal.pone.0010036 20418945 PMC2854682

[39] Dukic V, Lopes HF, Polson NG. Tracking Epidemics With Google Flu Trends Data and a State-Space SEIR Model. http://dx.doi.org/101080/016214592012713876. Taylor & Francis Group; 2012; 10.1080/01621459.2012.713876 PMC10426794 37583443

[40] Boraschi D, Aguado MT, Dutel C, Goronzy J, Louis J, Grubeck-Loebenstein B, et al The gracefully aging immune system. Sci Transl Med. 2013;5: 185ps8 10.1126/scitranslmed.3005624 23677590

[41] Fulton RB, Varga SM. Effects of aging on the adaptive immune response to respiratory virus infections. Aging health. 2009;5: 775 10.2217/ahe.09.69 20174457 PMC2822389

[42] Lee FE-H, Walsh EE, Falsey AR, Betts RF, Treanor JJ. Experimental infection of humans with A2 respiratory syncytial virus. Antiviral Res. 2004;63: 191–6. 10.1016/j.antiviral.2004.04.005 15451187

[43] Velasco-Hernández JX, Núñez-López M, Comas-García A, Cherpitel DEN, Ocampo MC. Superinfection between Influenza and RSV Alternating Patterns in San Luis Potosí State, México. Tang JW, editor. PLoS One. 2015;10: e0115674 10.1371/journal.pone.0115674 25803450 PMC4372574

[44] Moscona A. RSV vaccine: Beating the virus at its own game. Sci Transl Med. 2015;7: 312fs44 10.1126/scitranslmed.aad2515 26537253

