# Supplementary figures and images for: Retrospective Parameter Estimation and Forecast of Respiratory Syncytial Virus in the United States
Source: PLoS Comput Biol. 2016 Oct 7;12(10):e1005133. doi: 10.1371/journal.pcbi.1005133 (PMC5055361; doi:10.1371/journal.pcbi.1005133)

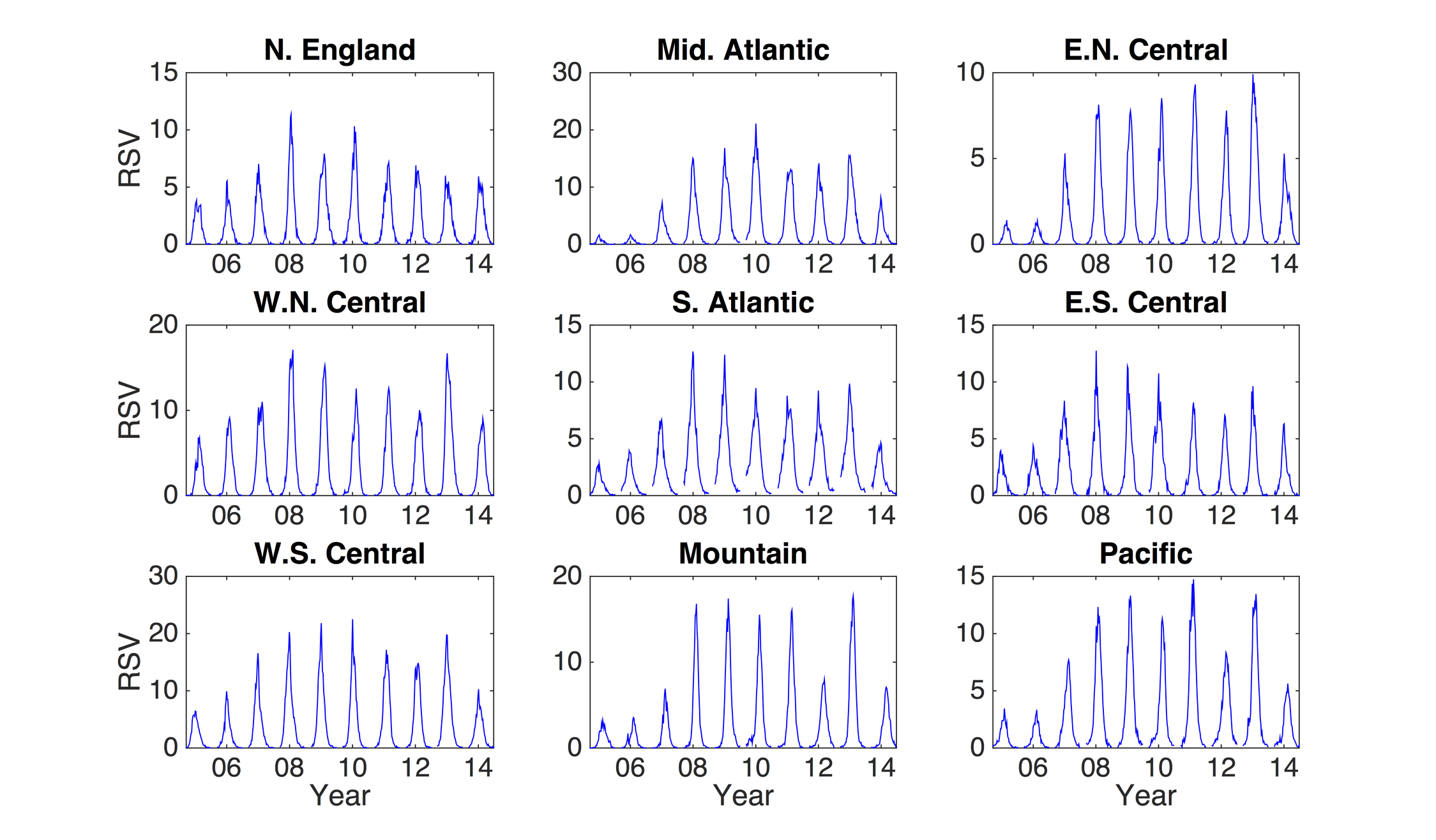

Supplement: S1 Fig — (TIF) [file pcbi.1005133.s001.tif]

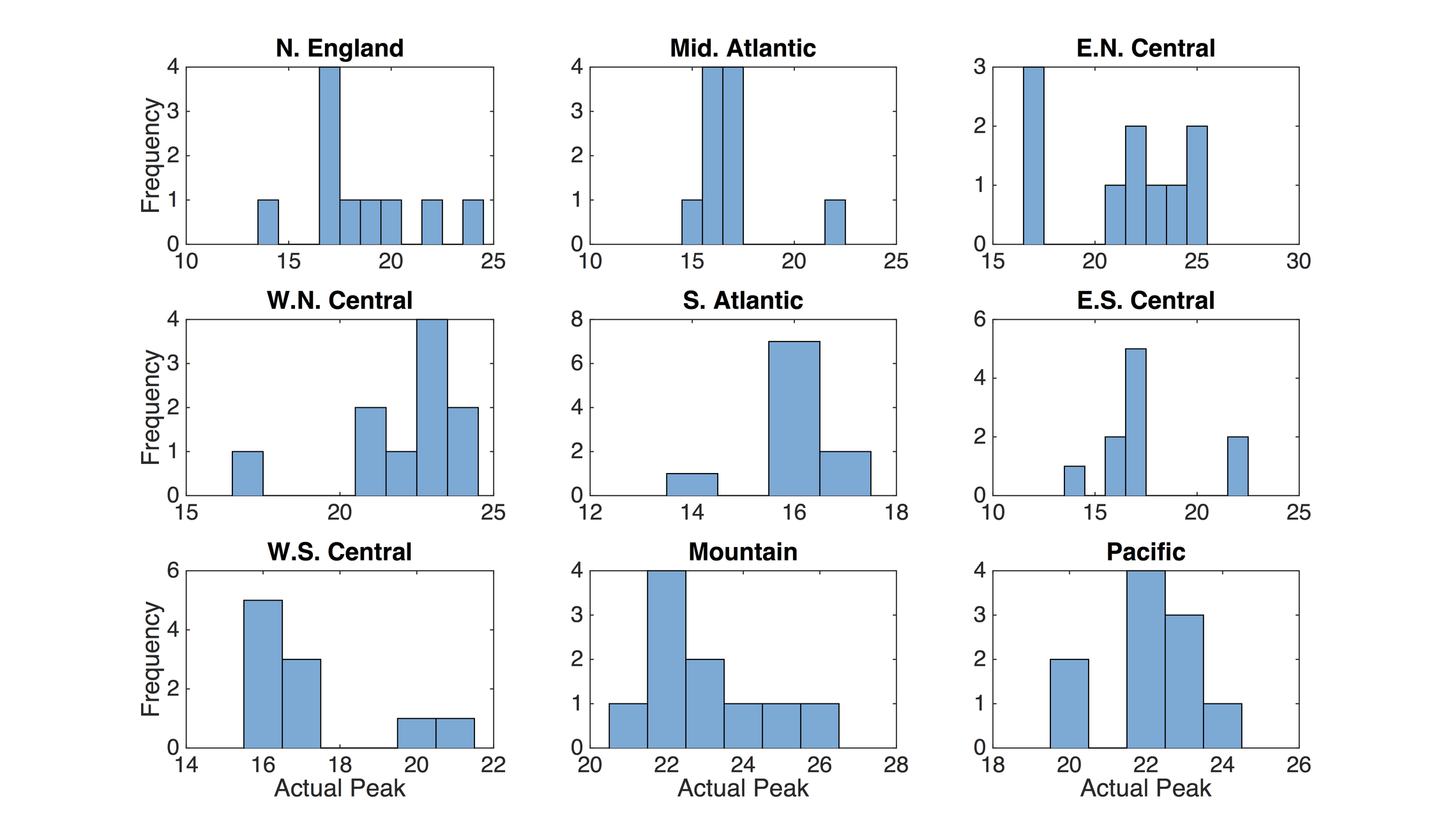

Supplement: S2 Fig — (TIF) [file pcbi.1005133.s002.tif]

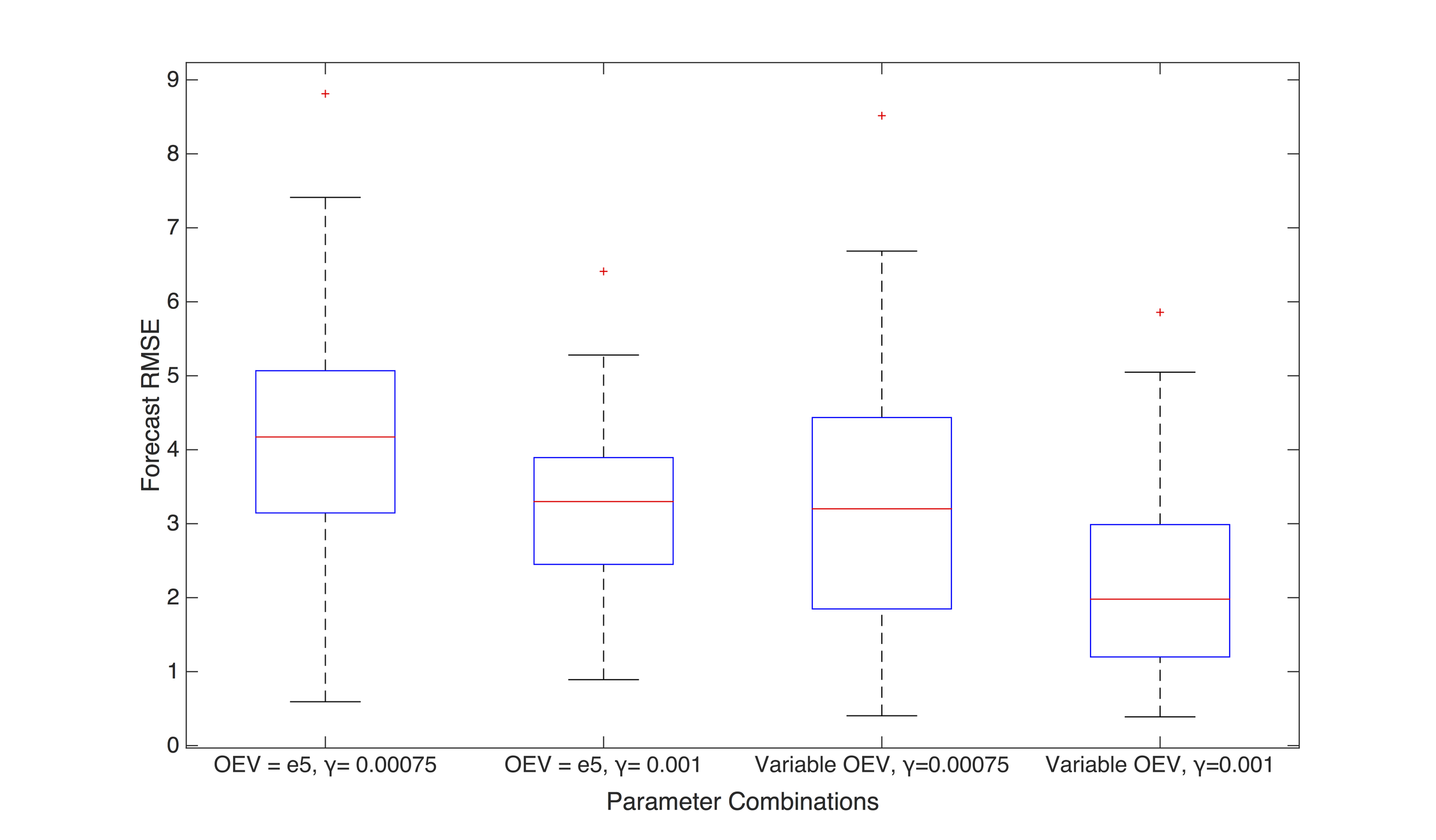

Supplement: S3 Fig — The variable OEV was computed with Eq 4, with OEV0 and a equal to 104 and 50, respectively. (TIF) [file pcbi.1005133.s003.tif]

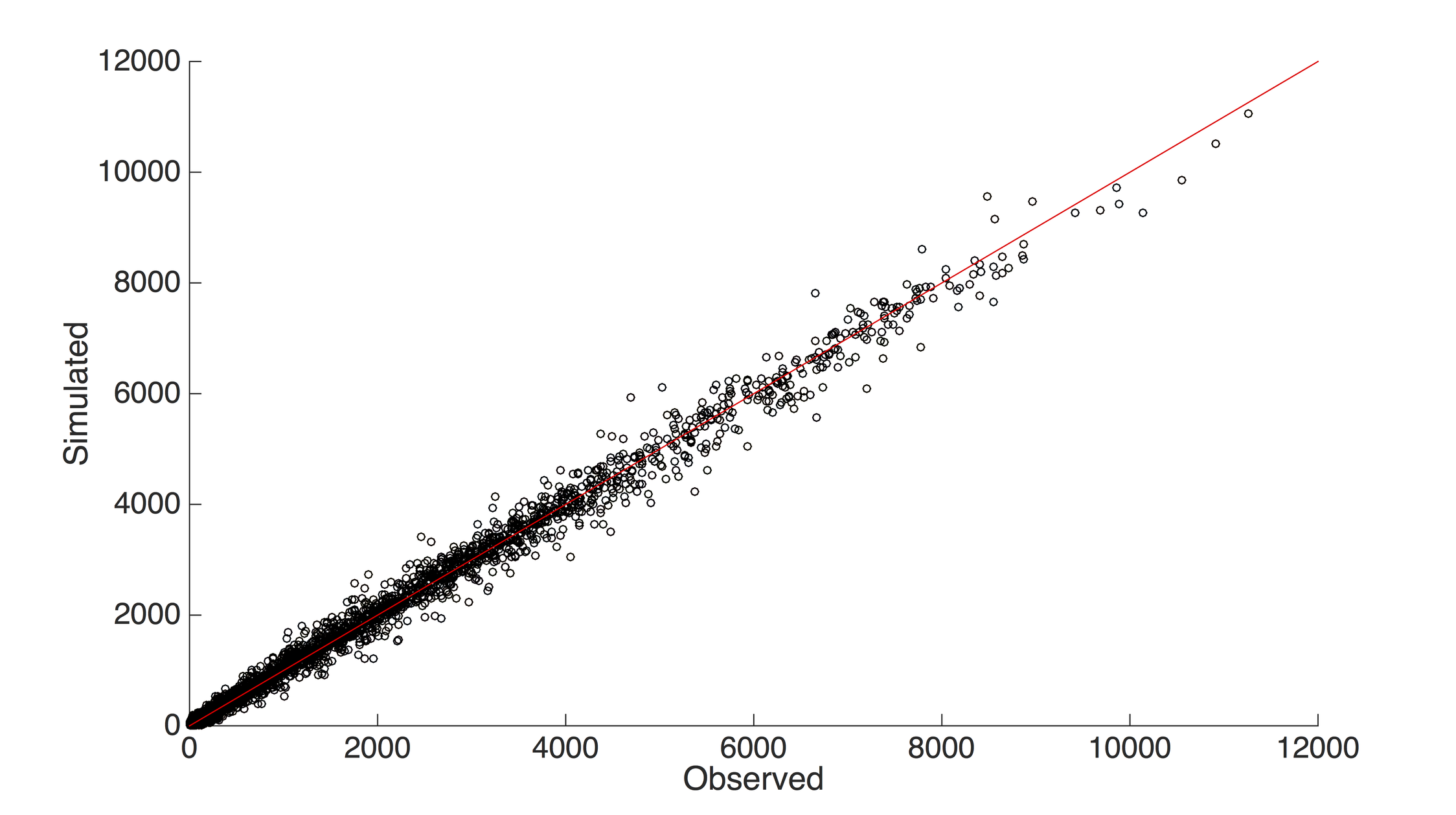

Supplement: S4 Fig — The red line is 1:1. (TIF) [file pcbi.1005133.s004.tif]

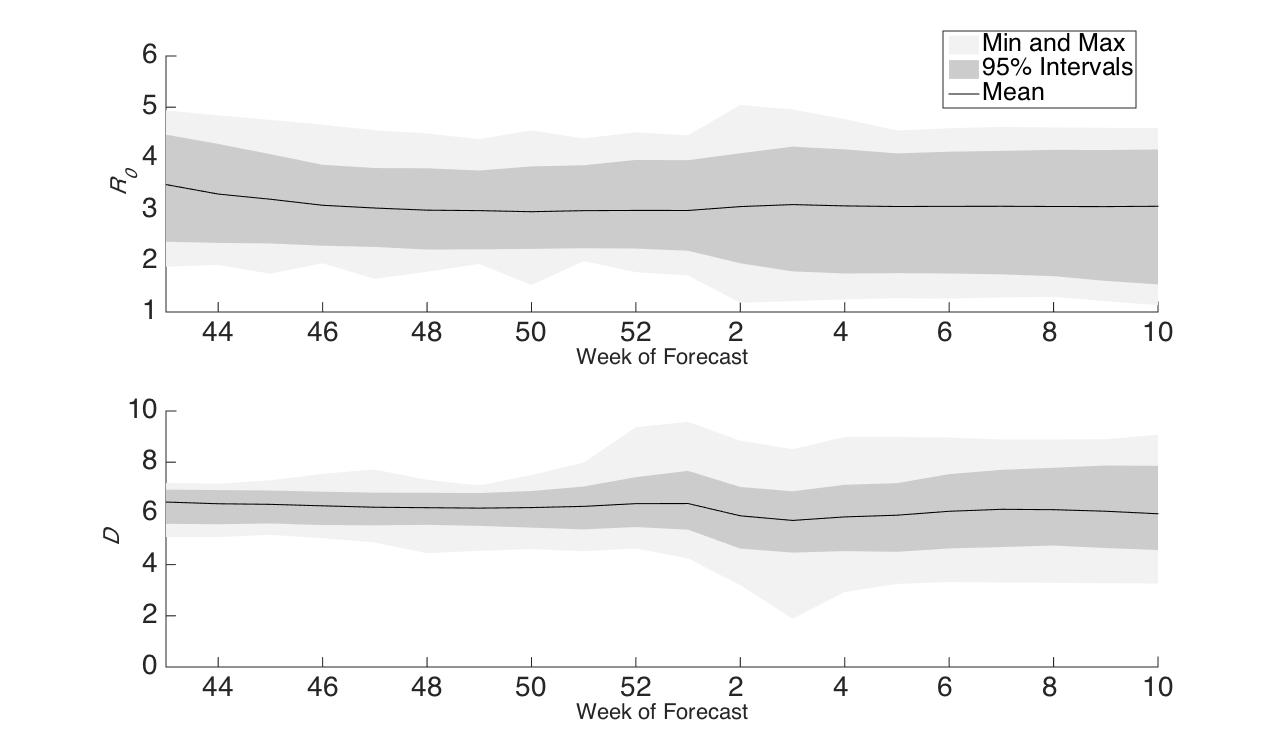

Supplement: S5 Fig — All regions and seasons are shown. (TIF) [file pcbi.1005133.s005.tif]

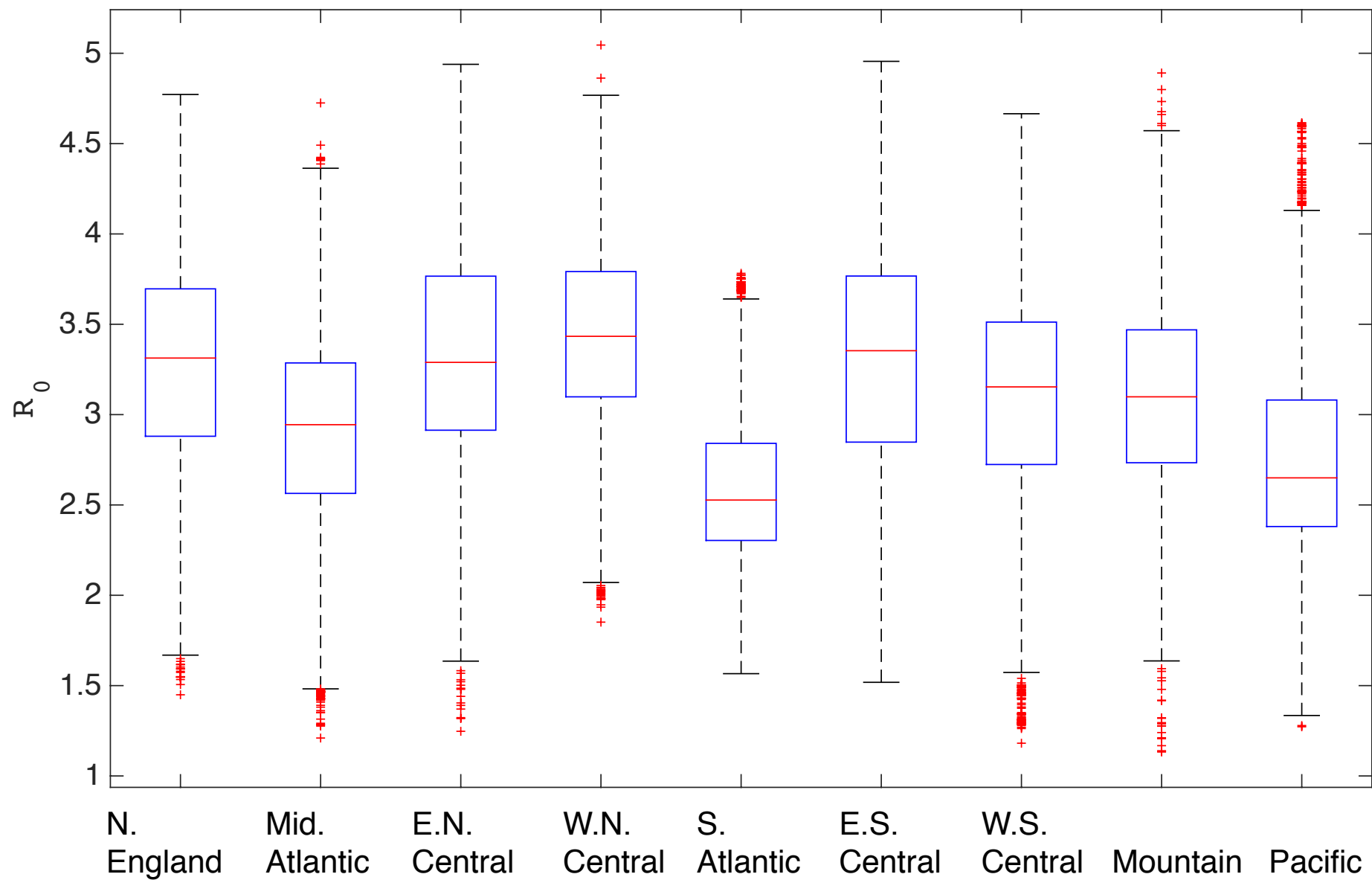

Supplement: S6 Fig — (PDF) [file pcbi.1005133.s006.pdf]

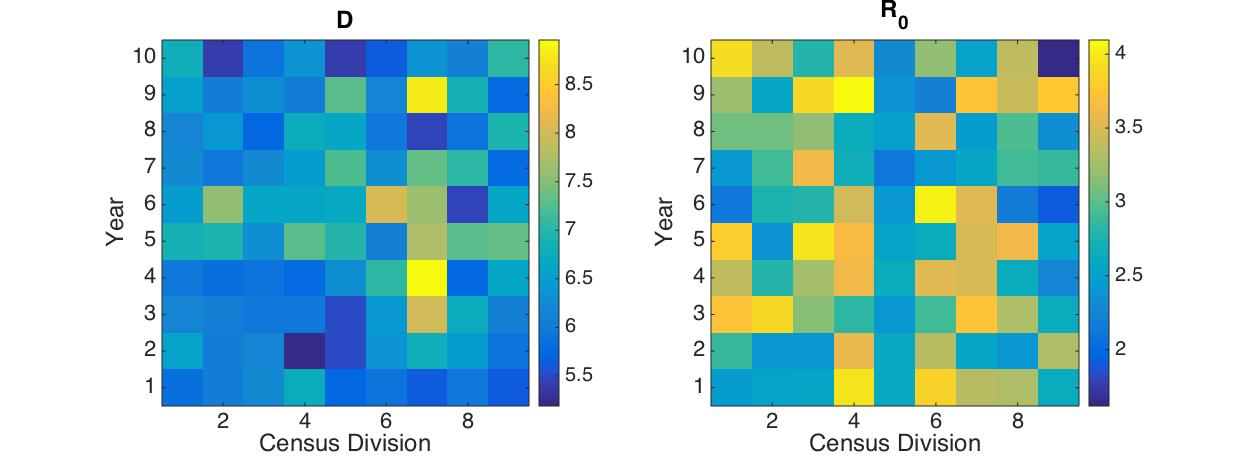

Supplement: S7 Fig — These estimates remain similar from year to year and location. Overall, the smallest mean R0 is 2.5, in Census Division 5, which contains Florida, and the largest mean R0 is 3.4 in Census Division 4. (TIF) [file pcbi.1005133.s007.tif]

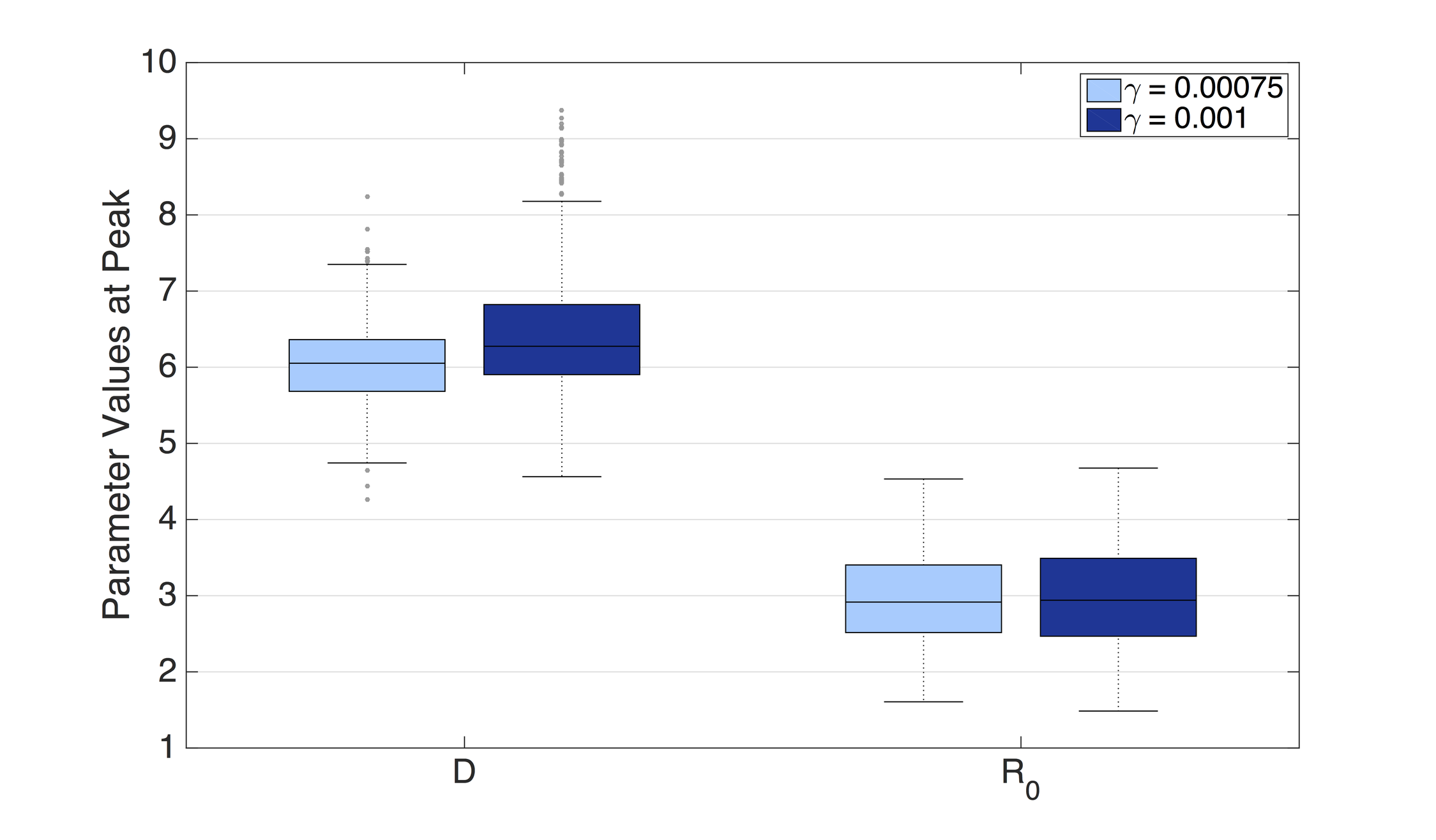

Supplement: S8 Fig — (TIF) [file pcbi.1005133.s008.tif]

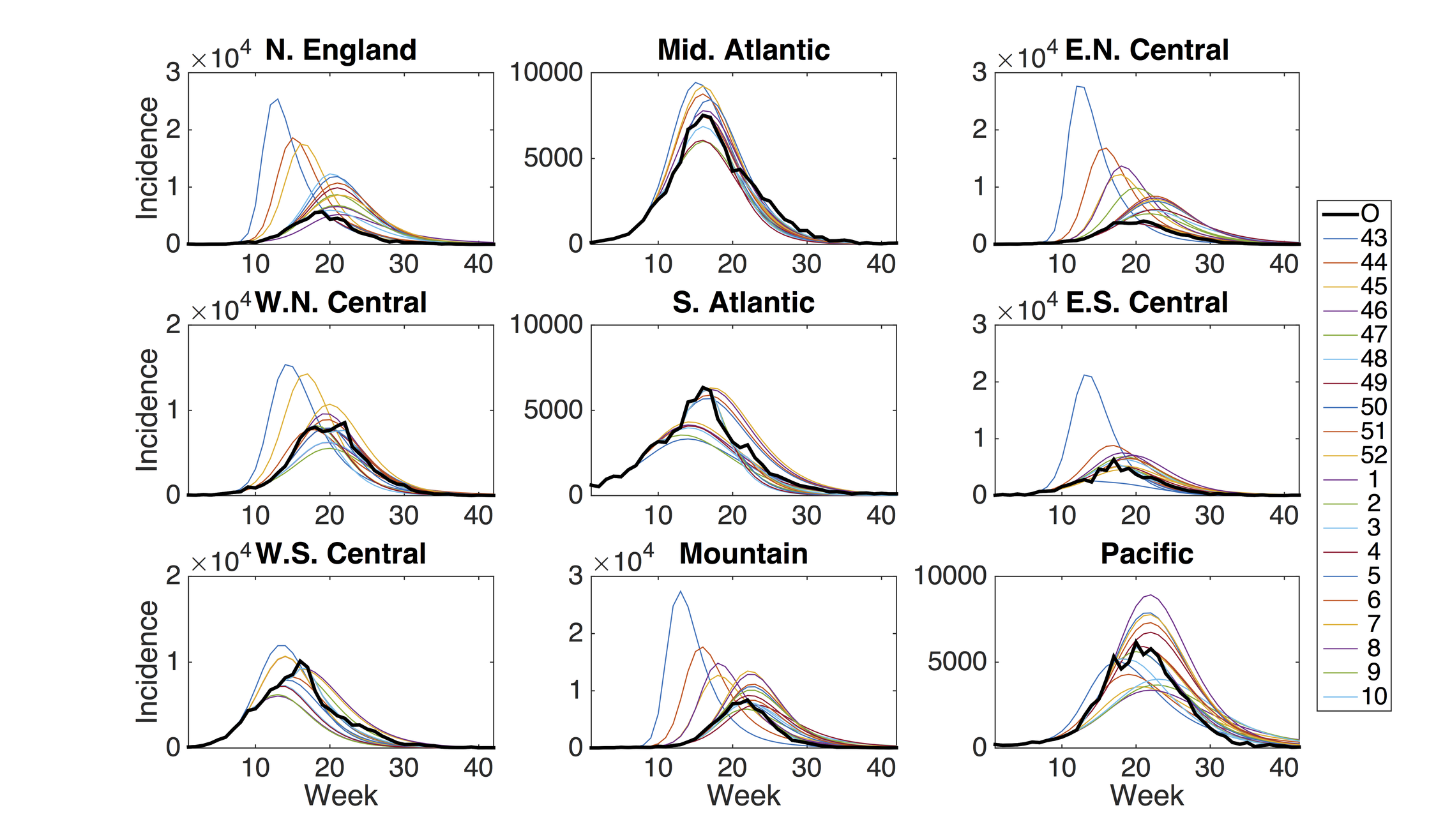

Supplement: S9 Fig — Each forecast shown is the mean of 300 ensemble members. The thick black line is observed RSV. The thin colored lines represent the ensemble mean trajectories of successive weekly forecasts, beginning Week 43 through Week 10. (TIF) [file pcbi.1005133.s009.tif]

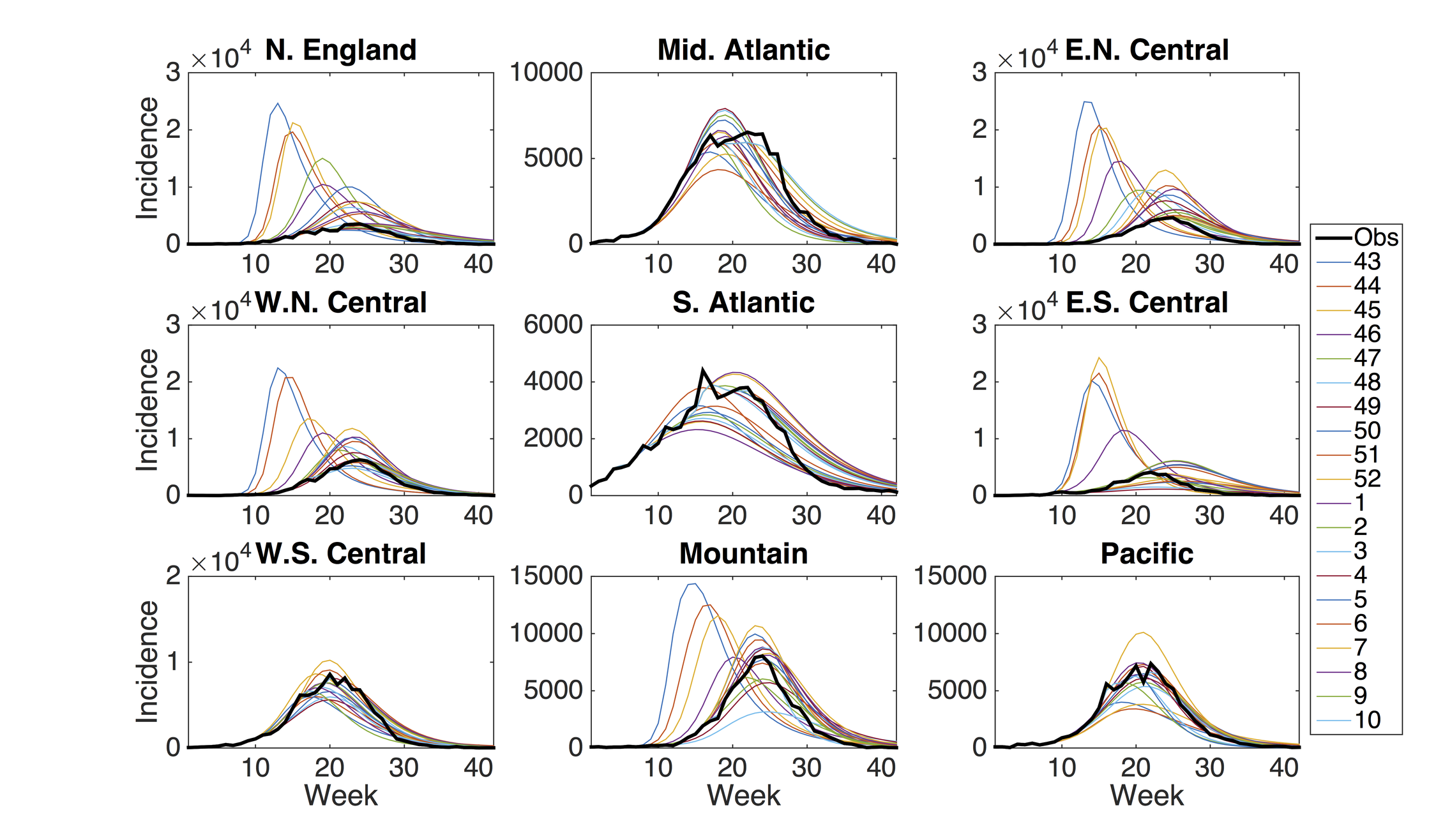

Supplement: S10 Fig — Each forecast shown is the mean of 300 ensemble members. The thick black line is observed RSV. The thin colored lines represent the ensemble mean trajectories of successive weekly forecasts, beginning Week 43 through Week 10. (TIF) [file pcbi.1005133.s010.tif]

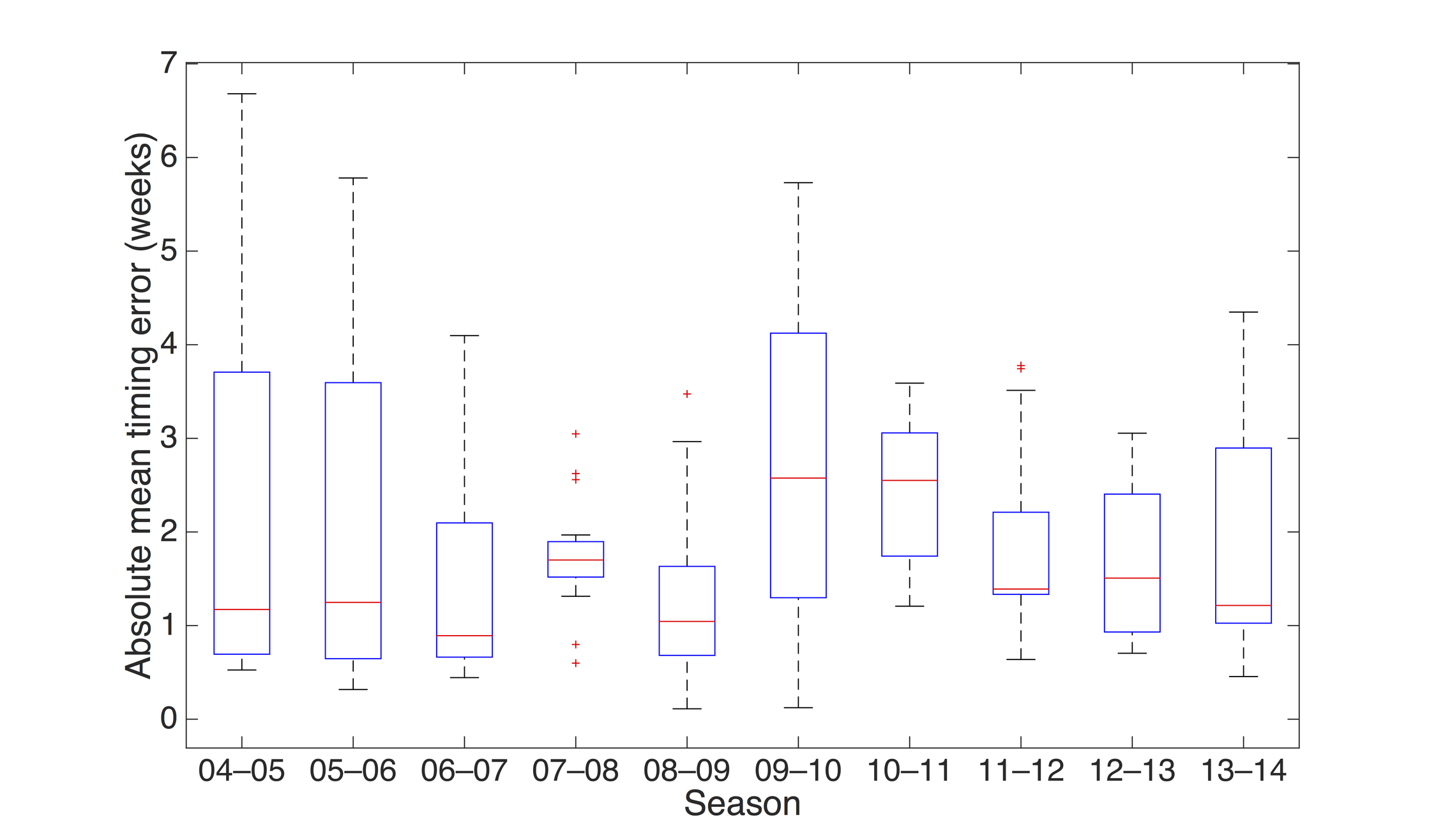

Supplement: S11 Fig — All forecasts, regardless of ensemble variance, are shown. (TIF) [file pcbi.1005133.s011.tif]

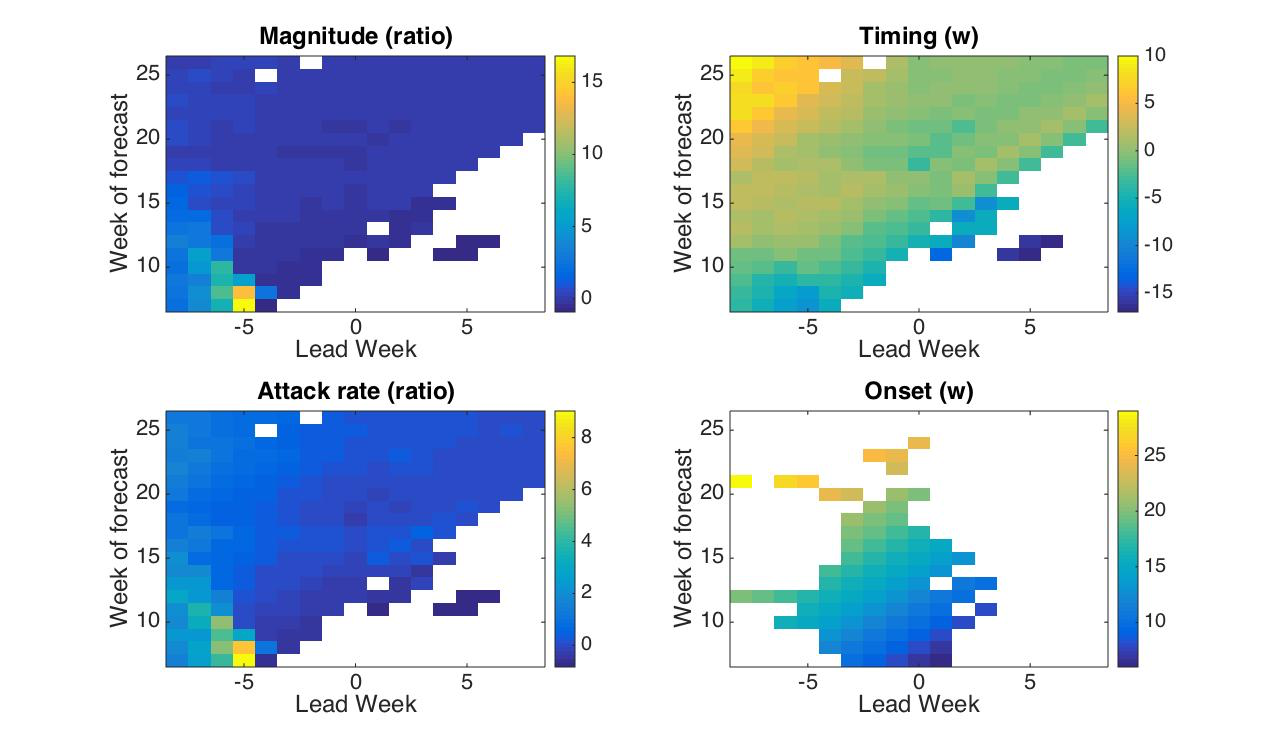

Supplement: S12 Fig — Blanks indicate week and lead week combinations without forecasts. Error decreases as both week of forecast and lead week increase. Including week of forecast along with ensemble variance and lead week could refine prediction calibration. (TIF) [file pcbi.1005133.s012.tif]

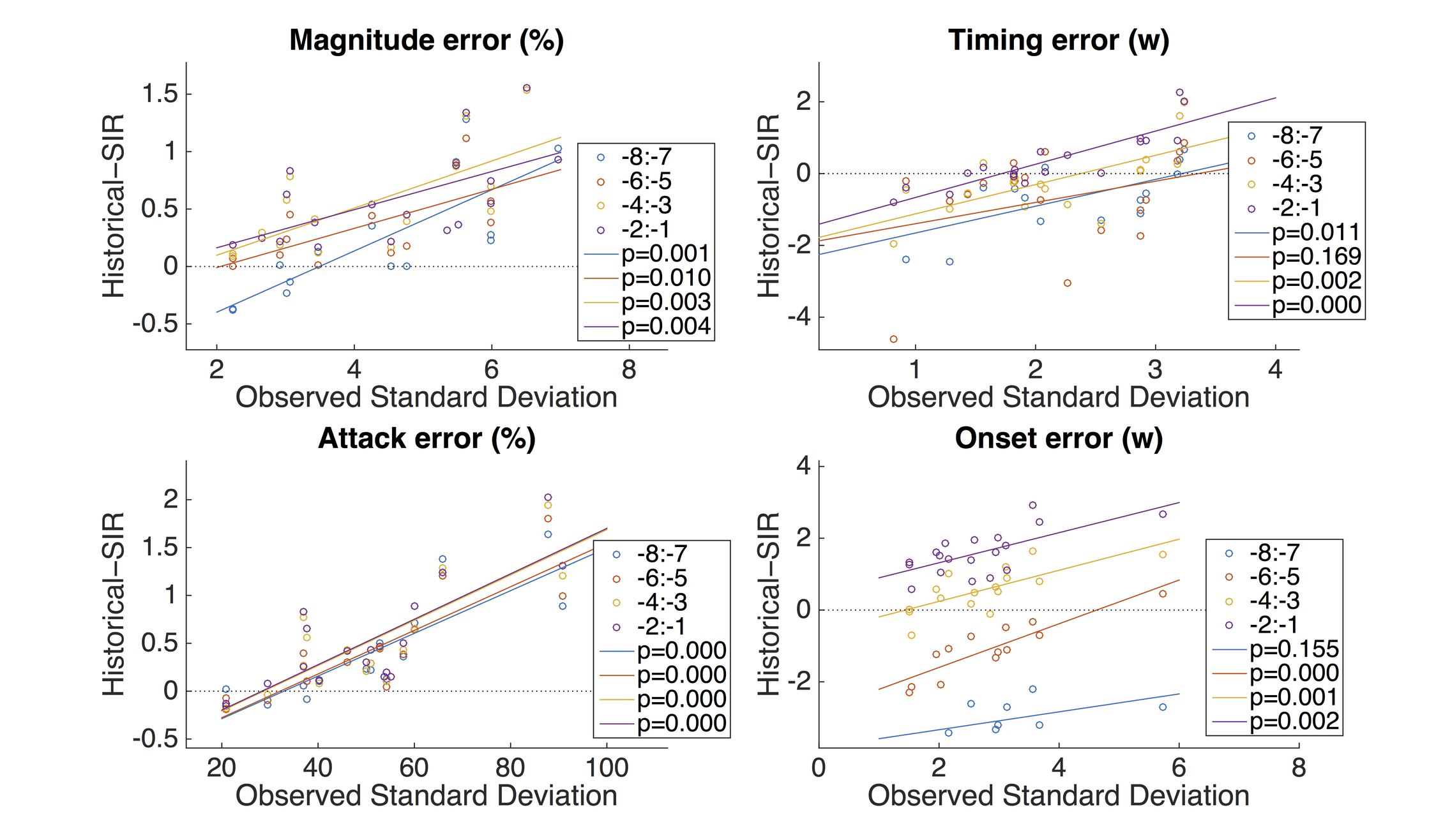

Supplement: S13 Fig — Scatter points above the zero line represent forecasts that outperform the historical mean. Both census division (CD) and HHS regions are plotted. Only forecasts with ensemble variance in the bottom 50th percentile, taken over the entire forecast period for each region, are shown; during some lead weeks, there are no forecasts in the bottom 50th percentile, hence some regions do not have all four lead week groupings (e.g. 7–8 weeks before the predicted onset). Positive significant linear correlations (alpha = 0.05) between difference in error and observed standard deviation are found for each forecast criterion at least four weeks in advance of the predicted peak or onset. S4 Table lists the regression line slope estimates for each regression and statistical significance. (TIF) [file pcbi.1005133.s013.tif]
