# Supplementary material for: Retrospective Parameter Estimation and Forecast of Respiratory Syncytial Virus in the United States
Source: PLoS Comput Biol. 2016 Oct 7;12(10):e1005133. doi: 10.1371/journal.pcbi.1005133 (PMC5055361; doi:10.1371/journal.pcbi.1005133)
Supplement: S1 Table — (DOCX) [file pcbi.1005133.s014.docx]

**S1 Table. US geographic partitions comprising each of the 10 US Department of Health and Human Services regions.**

| *HHS Region 1 (Boston): Connecticut, Maine, Massachusetts, New Hampshire, Rhode Island, and Vermont.* |
| --- |
| *HHS Region 2 (New York): New Jersey, New York, Puerto Rico, and the Virgin Islands.* |
| *HHS Region 3 (Philadelphia): Delaware, District of Columbia, Maryland, Pennsylvania, Virginia, and West Virginia.* |
| *HHS Region 4 (Atlanta): Alabama, Florida, Georgia, Kentucky, Mississippi, North Carolina, South Carolina, and Tennessee.* |
| *HHS Region 5 (Chicago): Illinois, Indiana, Michigan, Minnesota, Ohio, and Wisconsin.* |
| *HHS Region 6 (Dallas): Arkansas, Louisiana, New Mexico, Oklahoma, and Texas.* |
| *HHS Region 7 (Kansas City): Iowa, Kansas, Missouri, and Nebraska.* |
| *HHS Region 8 (Denver): Colorado, Montana, North Dakota, South Dakota, Utah, and Wyoming.* |
| *HHS Region 9 (San Francisco): Arizona, California, Hawaii, Nevada, American Samoa, Commonwealth of the Northern Mariana Islands, Federated States of Micronesia, Guam, Marshall Islands, and Republic of Palau.* |
| *HHS Region 10 (Seattle): Alaska, Idaho, Oregon, and Washington.* |
