# Supplementary material for: Retrospective Parameter Estimation and Forecast of Respiratory Syncytial Virus in the United States
Source: PLoS Comput Biol. 2016 Oct 7;12(10):e1005133. doi: 10.1371/journal.pcbi.1005133 (PMC5055361; doi:10.1371/journal.pcbi.1005133)
Supplement: S2 Table — (DOCX) [file pcbi.1005133.s015.docx]

**S2 Table. US states comprising each of the 9 US Census Divisions.**

| *Division 1 (New England): Connecticut, Maine, Massachusetts, New Hampshire, Rhode Island and Vermont.* |
| --- |
| *Division 2 (Middle Atlantic): New Jersey, New York and Pennsylvania.* |
| *Division 3 (East North Central): Indiana, Illinois, Michigan, Ohio and Wisconsin.* |
| *Division 4 (West North Central): Iowa, Kansas, Minnesota, Missouri, Nebraska, North Dakota and South Dakota.* |
| *Division 5 (South Atlantic): Delaware, District of Columbia, Florida, Georgia, Maryland, North Carolina, South Carolina, Virginia and West Virginia.* |
| *Division 6 (East South Central): Alabama, Kentucky, Mississippi and Tennessee.* |
| *Division 7 (West South Central): Arkansas, Louisiana, Oklahoma and Texas.* |
| *Division 8 (Mountain): Arizona, Colorado, Idaho, New Mexico, Montana, Utah, Nevada and Wyoming.* |
| *Division 9 (Pacific): Alaska, California, Hawaii, Oregon and Washington.* |
