# Supplementary material for: Retrospective Parameter Estimation and Forecast of Respiratory Syncytial Virus in the United States
Source: PLoS Comput Biol. 2016 Oct 7;12(10):e1005133. doi: 10.1371/journal.pcbi.1005133 (PMC5055361; doi:10.1371/journal.pcbi.1005133)
Supplement: S3 Table — (DOCX) [file pcbi.1005133.s016.docx]

**S3 Table. State variables and parameters estimated using the SIRS-EAKF system and the range of values employed for random initialization.**

| *Variable or parameter* | *Initialized Range* | *Source or justification* |
| --- | --- | --- |
| Susceptible, *S* | 1.4x10^5^ – 4.9x10^5^ people | Broad range |
| Infectious, *I* | One to 1.5x10^3^ people | Broad range |
| Duration of Infection, *D* | 4–9 days | [24], [25] |
| Reproductive number, *R_0_* | 2.8–8.2 new cases per infection | [21]–[23], [45] |
