# Supplementary material for: Retrospective Parameter Estimation and Forecast of Respiratory Syncytial Virus in the United States
Source: PLoS Comput Biol. 2016 Oct 7;12(10):e1005133. doi: 10.1371/journal.pcbi.1005133 (PMC5055361; doi:10.1371/journal.pcbi.1005133)
Supplement: S4 Table — Only forecasts with ensemble variance in the lowest 50th percentile were included. (DOCX) [file pcbi.1005133.s017.docx]

**S4 Table. Statistics for the difference between the historical regional mean and model-filter forecasts error regressed upon the observed standard deviation of each criterion (plotted in S12 Figure).** Only forecasts with ensemble variance in the lowest 50th percentile were included.

|  | **Lead Week** | **Magnitude** | **Timing** | **Attack Rate** | **Onset** |
| --- | --- | --- | --- | --- | --- |
| Slope | -8:-7 | 0.267 | 0.747 | 0.022 | 0.250 |
|  | -6:-5 | 0.171 | 0.593 | 0.023 | 0.609 |
|  | -4:-3 | 0.205 | 0.816 | 0.024 | 0.433 |
|  | -2:-1 | 0.166 | 0.925 | 0.024 | 0.419 |
| p slope | -8:-7 | 0.001 | 0.011 | 0.000 | 0.155 |
|  | -6:-5 | 0.010 | 0.169 | 0.000 | 0.000 |
|  | -4:-3 | 0.003 | 0.002 | 0.000 | 0.001 |
|  | -2:-1 | 0.004 | 0.000 | 0.000 | 0.002 |
| Intercept | -8:-7 | -0.931 | -2.401 | -0.733 | -3.834 |
|  | -6:-5 | -0.350 | -1.989 | -0.733 | -2.817 |
|  | -4:-3 | -0.313 | -1.938 | -0.680 | -0.625 |
|  | -2:-1 | -0.169 | -1.590 | -0.672 | 0.479 |
